# Supplementary material for: Effect of Dexamethasone on the Incidence and Outcome of COVID-19 Associated Pulmonary Aspergillosis (CAPA) in Critically Ill Patients during First- and Second Pandemic Wave—A Single Center Experience
Source: Diagnostics (Basel). 2022 Dec 5;12(12):3049. doi: 10.3390/diagnostics12123049 (PMC9777363; doi:10.3390/diagnostics12123049)
Supplement: Supplementary file 1 [file diagnostics-12-03049-s001.zip › diagnostics-2041384-Supplementary.pdf]

Supplementary Material

Table S1: Specific treatment for patients with COVID-19

	**DEXA (n=169)**	**Non-DEXA (n=62)**
Remdesivir	136	13
Hydroxychloroquin	0	21
Plasma Exchange	0	8
Sarilumab	0	3
